# Flexor Tendon Rupture Following Collagenase *Clostridium histolyticum* Versus Percutaneous Needle Fasciotomy in the Management of Dupuytren’s Disease: A Systematic Review and Meta-Analysis

**DOI:** 10.3390/jpm16090459

**Published:** 2026-08-31

**Authors:** Sherlyn Chng, Omar Shadid, Ishith Seth, Warren M. Rozen

**Affiliations:** 1 Department of Plastic and Reconstructive Surgery, Peninsula Health, Frankston, Melbourne 3199, Australia; sherlyn_chng02@hotmail.com (S.C.); warrenrozen@hotmail.com (W.M.R.); 2 Faculty of Medicine and Surgery, Peninsula Clinical School, Monash University, Frankston, Melbourne 3199, Australia

**Keywords:** Dupuytren’s disease, collagenase *Clostridium histolyticum*, percutaneous needle fasciotomy, flexor tendon rupture, meta-analysis, precision medicine, risk stratification

## Abstract

**Background:** Flexor tendon rupture is a rare but potentially disabling complication following minimally invasive treatment of Dupuytren’s disease. This meta-analysis compared rupture incidence after collagenase *Clostridium histolyticum* (CCH) injection and percutaneous needle fasciotomy/aponeurotomy (PNF/PNA). **Methods:** PubMed, MEDLINE, and Embase were systematically searched for randomised trials, cohort studies, clinical trials, and case series reporting complications after CCH or PNF for Dupuytren’s disease. Case reports and secondary evidence were excluded from incidence calculations, although reference lists of reviews were screened for eligible primary studies. Flexor tendon rupture events and study denominators were extracted as reported. Pooled incidence was estimated using random-effects proportional meta-analysis with logit transformation and continuity correction. Separate pooled estimates were generated for CCH and PNF, followed by subgroup comparison. **Results:** Forty-one studies were included. Across eligible studies, 68 flexor tendon ruptures were identified, including 62 after CCH and 6 after PNF. Ruptures most commonly involved the flexor digitorum profundus, affected the small finger, and occurred early, with a median time to rupture of 8 days, where reported. Fourteen PNF cohorts comprising 6058 treated units reported 6 ruptures, producing a pooled incidence of 0.32% (95% CI: 0.18–0.55%) with no observed heterogeneity. Twenty-nine CCH cohorts comprising 60,949 treated units reported 62 ruptures, producing a pooled incidence of 0.83% (95% CI: 0.42–1.64%) with substantial heterogeneity. Subgroup comparison demonstrated a higher pooled rupture incidence following CCH than PNF. **Conclusions:** Flexor tendon rupture is uncommon after both CCH and PNF. Characterisation of anatomical and treatment-related patterns of rupture may support more individualised risk counselling and treatment selection in Dupuytren’s disease. Future studies are required to develop clinically meaningful risk-stratification strategies.

## 1. Introduction

Dupuytren’s disease (DD) is a progressive fibroproliferative disorder of the palmar fascia, resulting in the formation of fibrous cords and subsequent flexion contractures of the fingers along the metacarpophalangeal (MP) and/or proximal interphalangeal (PIP) joints [1,2,3,4,5,6,7]. The aetiology is multifactorial and is not yet completely understood. However, it is thought that genetic predisposition, following an autosomal dominant pattern, as well as environmental factors such as manual labour and repetitive vibration exposure contribute to disease development [3].

Patients experience progressive flexion deformity, joint stiffness, and functional impairment of finger extension which reduces the quality of life for these patients [3].

Treatment options have evolved alongside advances in the understanding of DD pathophysiology. Minimally invasive treatments, including collagenase *Clostridium histolyticum* (CCH) injections and percutaneous needle fasciotomy (PNF—also termed PNA), have gained popularity due to shorter recovery times and generally favourable safety profiles compared to open surgery [2,5,6]. However, open surgery, more specifically limited fasciectomy, has been shown to have lower recurrence rates of Dupuytren’s contractures after treatment when compared with CCH or PNF [8,9,10].

The rationale behind which treatment to implement depends on a multitude of factors, including the degree of contracture, which joints are involved—historically, contracted proximal interphalangeal (PIP) joints are more resistant to correction compared to metacarpophalangeal (MCP) joints [6], the likelihood of recurrence post-treatment and the potential for complications [1,5].

PNF involves percutaneous division of the pathological cord using a needle, allowing passive extension of the affected digit [1,5,7]. In contrast, CCH is injected into the fibrotic cord, with enzymatic degeneration followed by manipulation of the digit 1 to 7 days later to achieve rupture of the cord [1,5].

Previous studies have shown that both PNF and CCH are comparable in their efficacy and complication risk, and so the decision behind which modality to use largely depends on patient and provider preference [7,8,9,10,11,12,13]. However, as with all medical treatments, PNF and CCH still come with their own risk of complications, although these are uncommon [1]. CCH injections are associated with a higher overall rate of complications [5,13,14]; however, most are minor and self-limiting [1,2,6,8], including pain, swelling, and bruising. Serious adverse events such as skin tears, flexor tendon ruptures or neurovascular injury are rare and reported to be less than 1% in total [1].

Flexor tendon ruptures may involve the flexor digitorum profundus (FDP) and/or flexor digitorum superficialis (FDS), either in isolation or in combination. The mechanism of injury differs between treatment modalities: PNF is associated with mechanical transection of the tendon, whereas CCH-related injury is thought to occur through enzymatic weakening and subsequent rupture. Previously reported rates of tendon rupture following minimally invasive treatments (CCH and PNF) fall between 0.05–0.3% [15,16,17,18], with potential risk factors including treatment of PIP joint contractures and the small finger, recurrent Dupuytren’s disease, and previous surgical treatment of contractures [19,20,21].

Given the heterogeneity of Dupuytren’s disease in anatomical distribution, contracture severity, previous treatment, and recurrence, treatment selection should account for individual patient and disease characteristics. Defining rare but clinically significant complications within specific anatomical and procedural contexts may therefore contribute to a more personalized approach to patient counselling and selection between minimally invasive treatment options.

Although previous systematic reviews have compared the broader efficacy, recurrence, and complication profiles of CCH and PNF, serious adverse events such as flexor tendon rupture have been too uncommon within direct comparative studies for meaningful quantitative analysis. The present review therefore focuses specifically on flexor tendon rupture and incorporates a broader range of primary evidence, including single-arm cohorts and post-marketing surveillance studies, to estimate its incidence following CCH and PNF. This rare-event-focused approach addresses a clinically important complication that has not been the primary focus of previous comparative reviews.

Therefore, this systematic review and meta-analysis aims to evaluate and compare the incidence of flexor tendon ruptures following the use of PNF and CCH.

## 2. Methods

This systematic review was conducted in accordance with the Preferred Reporting Items for Systematic Reviews and Meta-Analyses (PRISMA). Initial screening of the literature identified a few studies specifically evaluating flexor tendon rupture following treatment of DD. Given the limited number of direct comparative studies between collagenase CCH and PNF, a comparative synthesis of flexor tendon rupture incidence data from separate study cohorts was performed.

A structured search of PubMed, MEDLINE and Embase was conducted to identify relevant randomised controlled trials (RCTs), cohort studies, and case series reporting complications associated with CCH or PNF. This systematic review was not registered.

### 2.1. Search Terms

A systematic search of PubMed, MEDLINE and Embase was performed using controlled vocabulary (MeSH/Emtree) and free-text terms. Search terms included “Dupuytren’s contracture”, “Dupuytren disease”, “collagenase clostridium histolyticum”, “CCH”, “collagenase injection”, “Xiaflex”, “percutaneous needle fasciotomy”, “percutaneous needle aponeurotomy”, “PNF”, “PNA” and “flexor tendon rupture”. Boolean operators (AND/OR) were used to combine concepts. Given the low frequency with which tendon rupture is reported as a primary outcome, an additional broader search using “adverse effect*” and “complication*” was performed to capture studies reporting relevant complications.

### 2.2. Inclusion and Exclusion Criteria

Studies were eligible if they reported primary clinical data from patients with Dupuytren’s disease treated with CCH and/or PNF/PNA and provided sufficient information to determine whether flexor tendon rupture occurred, including a treatment denominator from which incidence could be estimated. Eligible study designs included randomised controlled trials, non-randomised clinical trials, prospective and retrospective cohort studies, and case series. Studies reporting CCH or PNF alongside another intervention were eligible where data relating specifically to the CCH or PNF cohort could be extracted separately.

Studies were excluded if they involved animal or cadaveric models, did not report primary clinical outcome data, did not provide an extractable CCH or PNF treatment cohort, or were narrative reviews, systematic reviews, meta-analyses, instructional articles, conference abstracts, editorials or commentaries. Case reports were excluded from quantitative incidence calculations because a meaningful treatment denominator could not be established, although relevant mechanistic and clinical observations were considered qualitatively. Studies evaluating open fasciectomy or fasciotomy alone were excluded from the quantitative analysis.

### 2.3. Search Strategy

The initial search identified 228 studies. After duplicate removal, titles and abstracts were screened for relevance. Two independent reviewers (SC and OS) conducted title and abstract screening using Covidence, with discrepancies resolved by consensus or, where required, consultation with a third reviewer (IS). Full-text screening was also performed independently by SC and OS, with conflicts resolved by the senior reviewer (IS).

**Figure 1:**
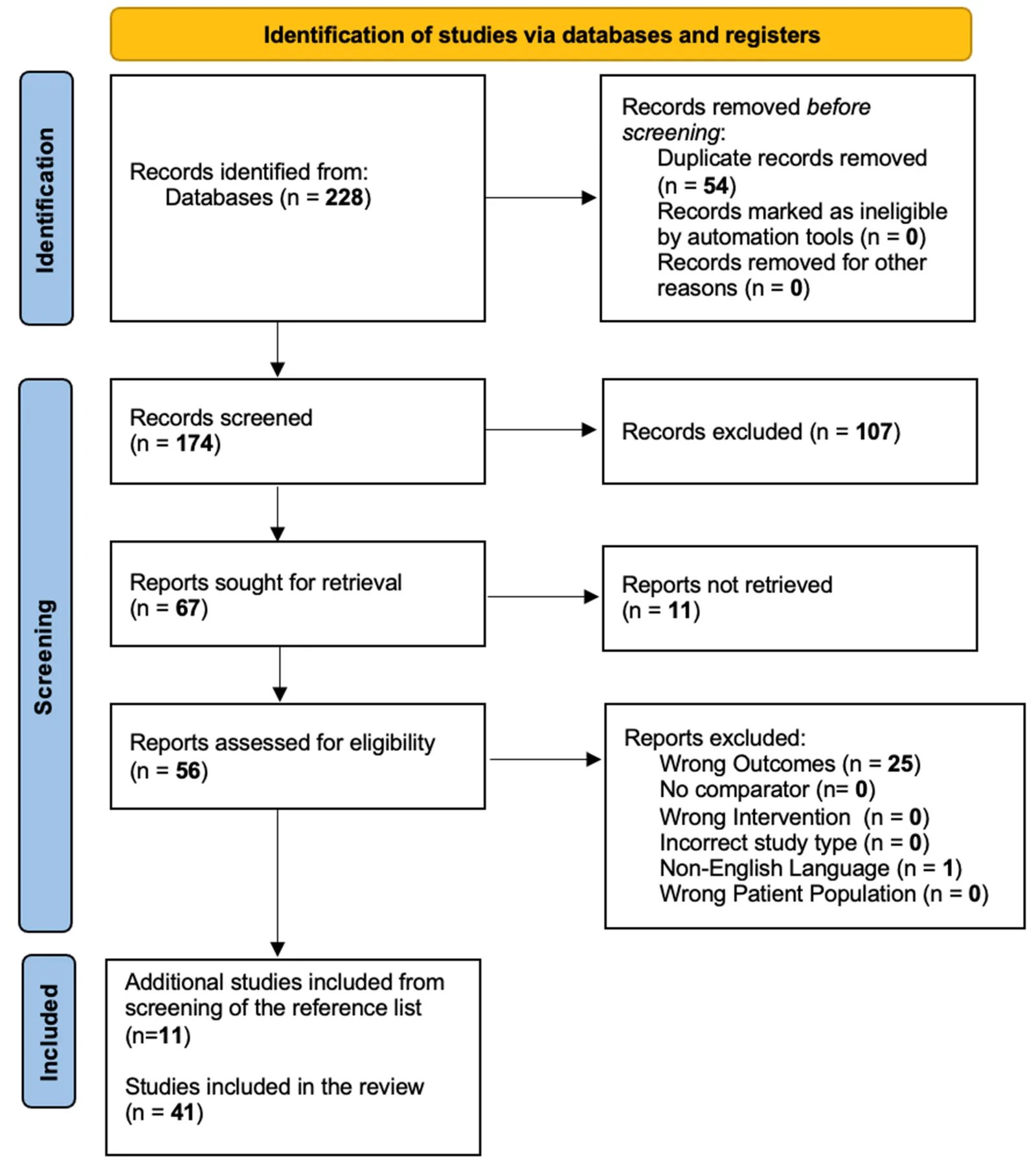
PRISMA flow diagram of reviewed articles.

Reference lists of relevant systematic reviews and meta-analyses, which were treated as secondary evidence, were screened to identify additional eligible primary studies. All citations were managed using Zotero. The study identification, screening and inclusion process is summarized in the PRISMA flow diagram (Figure 1).

Data was extracted using a standardised data-collection framework. Extracted variables included study design, treatment modality, sample size, patient demographics where available, number of flexor tendon ruptures, affected digit and joint, tendon involved (FDP, FDS or both), time from intervention to rupture, management, and reported clinical outcome. Denominator data were recorded using the unit reported in the original publication, including patients, fingers/digits, rays, or treatments, as consistent standardisation was not possible across studies. Digits/fingers were prioritised as the preferred denominator because flexor tendon rupture is a digit-level complication. Where digit or finger numbers were unavailable, the number of patients was used, followed by the number of treatments administered. When patient number was used, the total treated cohort was included, including patients lost to follow-up. Potentially overlapping study populations were assessed by comparing study centre, recruitment period, treatment modality, and cohort characteristics to minimise duplicate inclusion of the same patient population within the quantitative analysis.

### 2.4. Risk of Bias Assessment

Risk of bias was assessed using validated appraisal tools according to study design (Table 1). RCTs and randomised clinical trials were assessed using the Cochrane Risk of Bias Tool (RoB 2). Cohort studies, including retrospective, prospective, propensity score-matched, open-label and non-randomised clinical studies, were assessed using the Newcastle-Ottawa Scale (NOS). Case series were assessed using the Joanna Briggs Institute (JBI) Critical Appraisal Checklist for Case Series. Postmarketing safety analyses were evaluated narratively because their methodology did not fit NOS criteria.

### 2.5. Meta-Analysis

All statistical analyses and figure generation were performed in Python 3. For each study, the number of flexor tendon ruptures and the reported denominator were extracted directly from the included studies. Because tendon rupture was a rare event and many studies reported zero events, pooled incidence was estimated using a random-effects meta-analysis of proportions with a logit transformation and continuity correction. Specifically, for each study the observed proportion was stabilized using a continuity correction of 0.5 events and 1 added to the denominator before transformation to the logit scale. Within-study variance was calculated from the transformed proportions.

Between-study heterogeneity was modelled with a random-effects approach using the DerSimonian–Laird estimator for τ^2^. The pooled estimate and corresponding 95% confidence interval were then back-transformed to the original proportion scale and reported as incidence percentages. Statistical heterogeneity was summarized using Cochran’s Q and I^2^.

Separate meta-analyses were performed for CCH and PNF. A subgroup analysis comparing CCH and PNF was performed by comparing the pooled logit-transformed incidence estimates between groups using the standard error of the pooled subgroup effects.

Study-level confidence intervals displayed in the forest plots were calculated using exact binomial 95% confidence intervals. Forest plots were generated programmatically in Matplotlib v3.11.1 using a custom layout that aligned study labels, event counts, denominators, individual study estimates, confidence intervals, study weights, and pooled random-effects diamonds.

## 3. Results

### 3.1. Screening Outcomes

The search identified 228 studies across PubMed, MEDLINE and Embase. Following duplicate removal, title and abstract screening, and full-text review, 37 studies met eligibility criteria (Figure 1). These comprised 30 primary studies, including cohort studies, RCTs, case series and clinical trials, and 7 secondary studies, including systematic reviews, meta-analyses, literature reviews and clinical trial analyses.

The 7 secondary studies were excluded from primary data extraction; however, their reference lists were screened for additional eligible studies. This identified a further 11 primary studies, resulting in 41 studies included in the final review.

### 3.2. Risk of Bias Assessment

Overall methodological quality was moderate. RCTs generally demonstrated a low risk of bias, whereas most cohort studies were rated as having a moderate risk, largely due to retrospective design and inconsistent outcome reporting. Case series assessed using the JBI checklist were also predominantly of moderate quality. Full table with scoring can be found in Supplementary Table S1.

### 3.3. Characteristics of Included Studies

Of the 41 included studies, 20 reported outcomes following CCH injection, 8 reported outcomes following PNF, and 8 directly compared CCH with PNF (Table 1). Additional comparative studies involving CCH or PNF versus limited or open fasciectomy were included where adverse event data for CCH or PNF could be extracted. Overall, 55 procedure groups were identified, including 33 CCH groups, 17 PNF groups, 3 fasciectomy groups and 2 placebo groups. For full list of studies and their associated data, please see Supplementary Table S2.

The total treated cohort for CCH and PNF comprised 7708 patients across 49 procedure groups, excluding fasciectomy/placebo groups and studies that did not report patient numbers. This included 3649 CCH patients and 4059 PNF patients. Peimer et al. [20] was excluded from patient-count calculations because it reported CCH treatments rather than patient numbers. Included cohorts were predominantly older and male, with a mean reported age of 65.5 years (SD 2.4). Follow-up varied substantially, with a mean duration of 19.6 months (SD 26.0).

Across the 41 included studies, 10 reported flexor tendon rupture as a complication of either CCH or PNF (Table 2). No flexor tendon ruptures were reported in the direct comparative CCH-versus-PNF studies.

A total of 68 flexor tendon ruptures were identified, of which 62 occurred following CCH and 6 following PNF. Reported rupture incidence ranged from 0.09% to 1.7% following CCH and from 0.12% to 0.21% following PNF. Among cases with tendon type reported, isolated FDP rupture was most common (*n* = 16), followed by combined FDP and FDS rupture (*n* = 7) and isolated FDS rupture (*n* = 4).

The MCPJ was the most frequently reported involved joint (*n* = 23), followed by the PIPJ (*n* = 20), with both MCPJ and PIPJ involvement reported in 3 cases. The small finger was most commonly affected (*n* = 32), followed by the ring finger (*n* = 12). Among cases with timing reported, rupture most often occurred within 2 weeks, with a median time to rupture of 8 days. Four delayed ruptures were reported after CCH injection, occurring at 3 months, 106 days, 112 days and 4 months. Most cases with reported management were treated surgically (*n* = 11), including tendon excision, tendon grafting, reconstruction and tenorrhaphy.

### 3.4. Meta Analysis

#### 3.4.1. PNF Cohort

**Figure 2:**
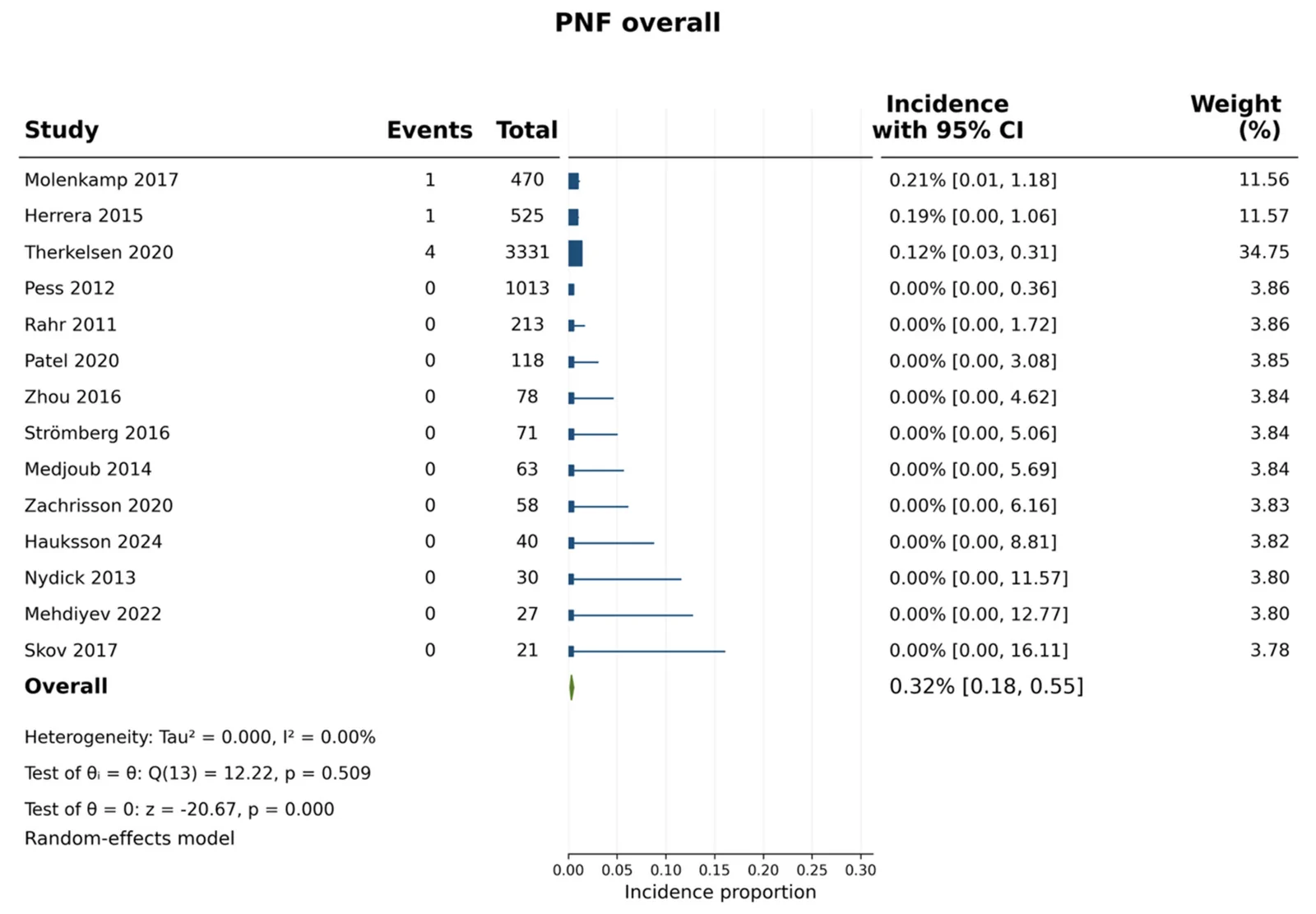
Pooled incidence of flexor tendon rupture following percutaneous needle fasciotomy/needle aponeurotomy. Forest plot showing the pooled incidence of flexor tendon rupture following percutaneous needle fasciotomy (PNF) for Dupuytren’s disease. Individual study estimates are presented as incidence proportions with 95% confidence intervals. Square size reflects study weighting in the random-effects model, and the diamond represents the pooled estimate. Across 14 PNF cohorts, the pooled incidence of flexor tendon rupture was 0.32% (95% CI: 0.18–0.55%). No statistical heterogeneity was observed between studies (τ^2^ = 0.000; I^2^ = 0.0%; Q(13) = 12.22, *p* = 0.509) [7,11,12,17,18,22,23,24,25,26,27,28,29].

Fourteen PNF cohorts were included in the pooled analysis, comprising 6058 treated fingers, digits, rays, or patients, depending on the denominator reported by each study (Figure 2). Six flexor tendon ruptures were reported across these cohorts.

Individual study-level incidence ranged from 0% to 0.21%, with rupture events reported by Herrera et al. [24], Molenkamp et al. [25], and Therkelsen et al. [7]. The pooled random-effects incidence of flexor tendon rupture following PNF was 0.32% (95% CI: 0.18–0.55%). There was no observed statistical heterogeneity between studies (τ^2^ = 0.000; I^2^ = 0.0%), and the test for heterogeneity was non-significant (Q(13) = 12.22, *p* = 0.509).

#### 3.4.2. CCH Cohort

**Figure 3:**
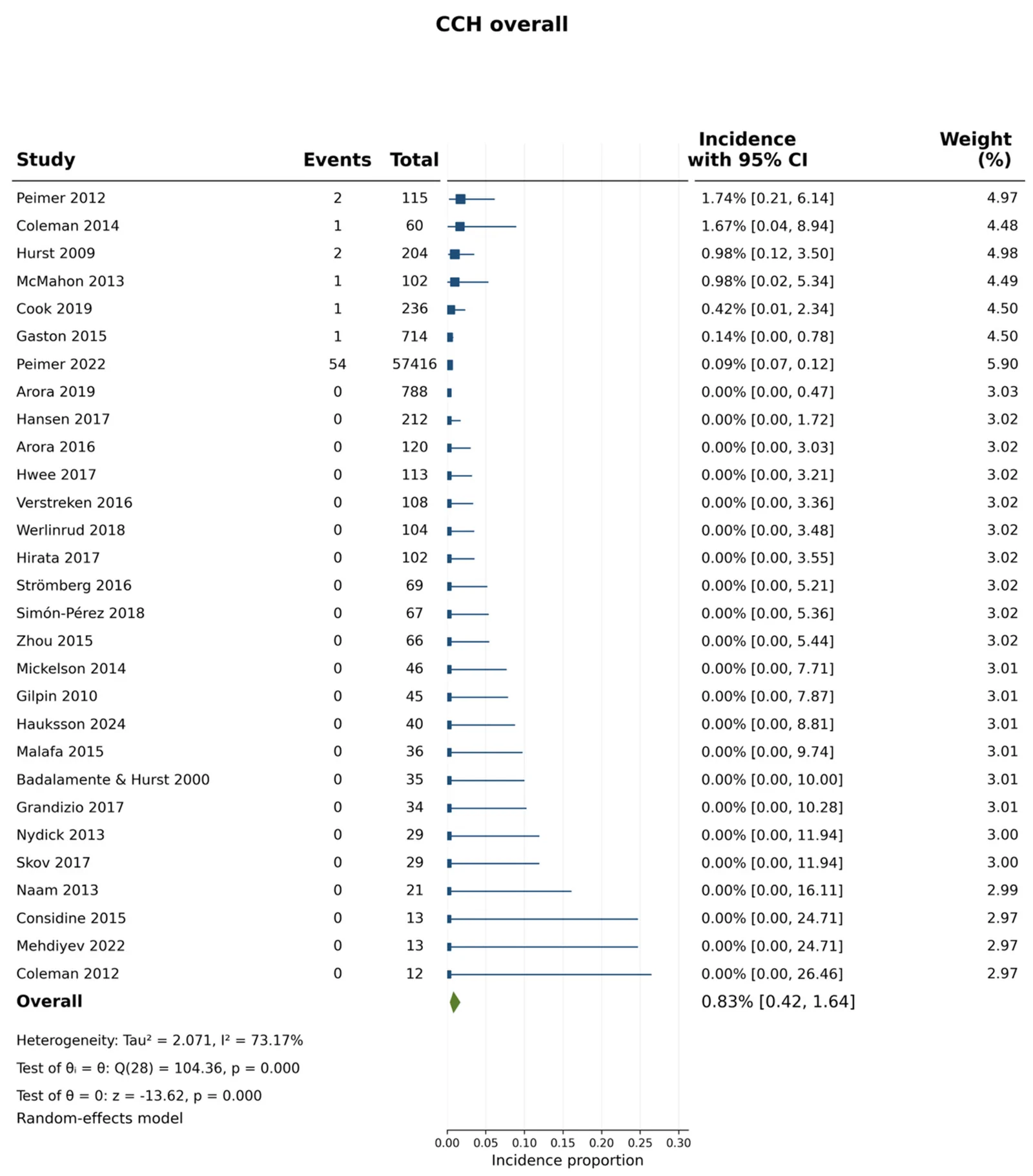
Pooled incidence of flexor tendon rupture following collagenase *Clostridium histolyticum* injection. Forest plot showing the pooled incidence of flexor tendon rupture following CCH injection for Dupuytren’s disease. Individual study estimates are presented as incidence proportions with 95% confidence intervals. Square size reflects study weighting in the random-effects model, and the diamond represents the pooled estimate. Across 29 CCH cohorts, the pooled incidence of flexor tendon rupture was 0.83% (95% CI: 0.42–1.64%). Substantial heterogeneity was observed between studies (τ^2^ = 2.071; I^2^ = 73.17%; Q(28) = 104.36, *p* < 0.001) [8,11,12,20,22,30,31,32,33,34,35,36,37,38,39,40,41,42,43,44,45,46,47,48,49,50,51,52,53].

Twenty-nine CCH cohorts were included in the pooled analysis, comprising 60,949 treated patients, digits, fingers, or treatments. Sixty-two flexor tendon ruptures were reported across these cohorts. Individual study-level incidence ranged from 0% to 1.74%, with the highest reported incidences observed in Peimer et al. 2012 [30], Coleman et al. 2014 [31], Hurst et al. 2009 [8], and McMahon et al. 2013 [32] (Figure 3). The large post-marketing safety analysis by Peimer et al. [20] contributed 54 rupture events from approximately 57,416 CCH treatments; however, it had a low study-level incidence of 0.09%.

The pooled random-effects incidence of flexor tendon rupture following CCH was 0.83% (95% CI: 0.42–1.64%). Substantial heterogeneity was observed among CCH studies (τ^2^ = 2.071; I^2^ = 73.17%), with a significant test for heterogeneity (Q(28) = 104.36, *p* < 0.001). This heterogeneity likely reflects differences in study design, denominator type, follow-up duration, adverse-event ascertainment, and the inclusion of post-marketing surveillance data.

#### 3.4.3. Subgroup Comparison Between CCH and PNF

**Figure 4:**
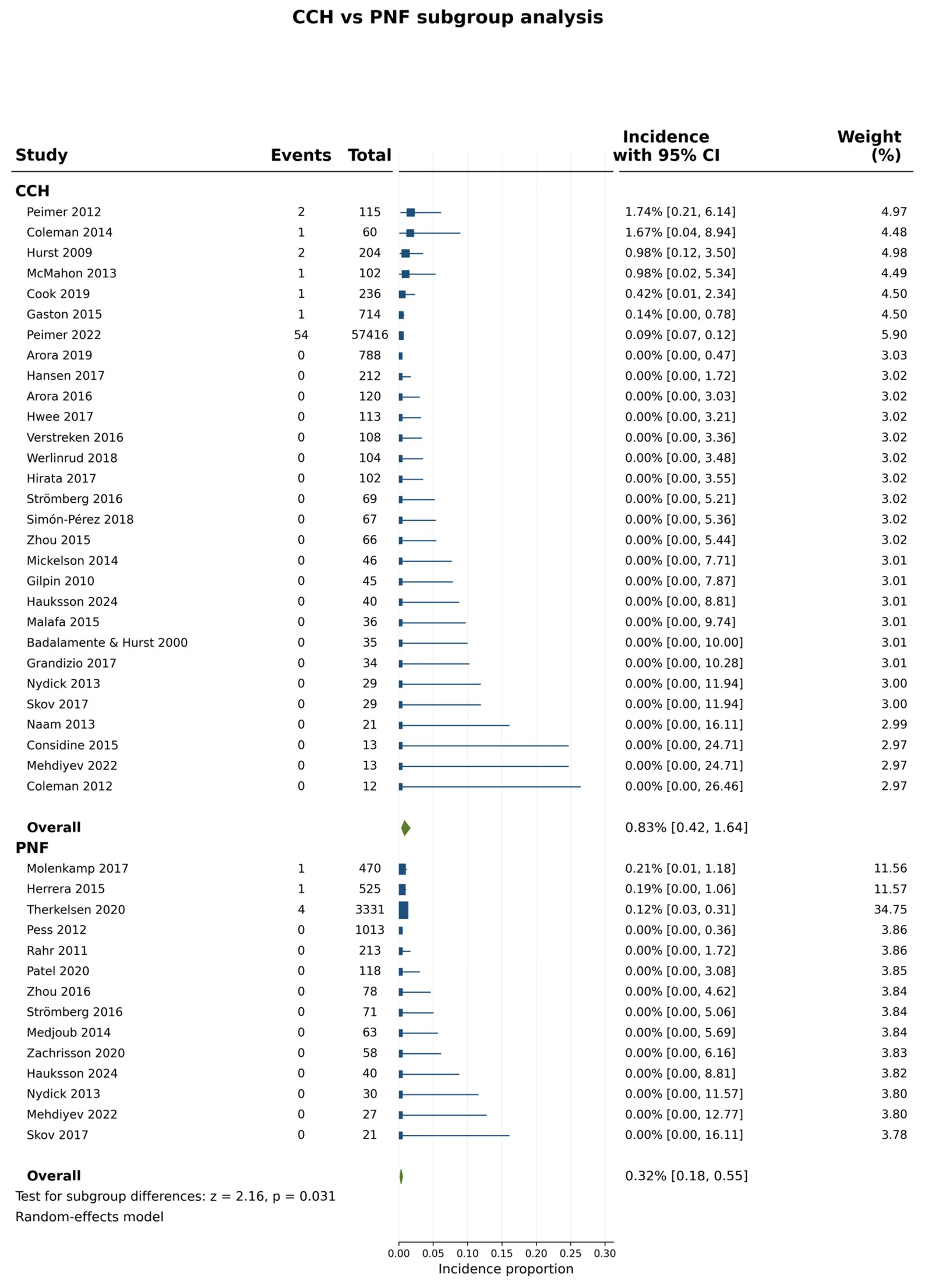
Subgroup comparison of flexor tendon rupture incidence following collagenase *Clostridium histolyticum* injection versus percutaneous needle fasciotomy/needle aponeurotomy. Forest plot comparing pooled flexor tendon rupture incidence between collagenase CCH injection and PNF for Dupuytren’s disease. Individual study estimates are presented as incidence proportions with 95% confidence intervals. Square size reflects study weighting, and diamonds represent pooled subgroup estimates from the random-effects model. The pooled incidence was higher following CCH than PNF, at 0.83% (95% CI: 0.42–1.64%) versus 0.32% (95% CI: 0.18–0.55%), respectively. The test for subgroup differences was statistically significant (z = 2.16, *p* = 0.031) [7,8,11,12,17,18,20,22,23,24,25,26,27,28,29,30,31,32,33,34,35,36,37,38,39,40,41,42,43,44,45,46,47,48,49,50,51,52].

The pooled incidence was 0.83% (95% CI: 0.42–1.64%) for CCH and 0.32% (95% CI: 0.18–0.55%) for PNF (Figure 4). The test for subgroup differences was statistically significant (z = 2.16, *p* = 0.031), indicating a difference between the pooled subgroup estimates.

Rupture characteristics demonstrated clinically relevant anatomical variation, with the small finger most frequently affected and FDP rupture the most commonly reported tendon injury where tendon involvement was specified. Timing was also variable, although most reported ruptures occurred early, with a median time to rupture of 8 days.

However, this finding should be interpreted cautiously. The analysis was based largely on separate single-arm cohorts rather than direct head-to-head comparative studies, and the included studies varied in their denominators, with some reporting patients, others fingers, digits, rays, or treatments. The uploaded report also notes that direct comparative inference is limited because comparative CCH-versus-PNF studies did not report rupture events.

Overall, flexor tendon rupture was an uncommon complication following both minimally invasive treatments for Dupuytren’s disease. The pooled incidence was higher following CCH than PNF, although interpretation is limited by sparse events, heterogeneous denominator reporting, and substantial heterogeneity among CCH studies.

## 4. Discussion

### 4.1. Flexor Tendon Rupture

This review and meta-analysis evaluated the incidence of flexor tendon rupture following minimally invasive treatment of DD, specifically CCH injection and PNF. Flexor tendon rupture was uncommon following both treatments; however, pooled incidence was higher after CCH than PNF.

In the random-effects proportional meta-analysis, the pooled incidence of flexor tendon rupture following CCH was 0.83% (95% CI: 0.42–1.64%), compared with 0.32% (95% CI: 0.18–0.55%) following PNF. Subgroup analysis demonstrated a statistically significant difference between treatment groups (z = 2.16, *p* = 0.031), suggesting a higher pooled rupture incidence following CCH. However, this finding should be interpreted cautiously because the analysis was primarily based on separate single-arm cohorts rather than direct comparative studies, and rupture events were sparse overall.

The CCH cohort demonstrated substantial heterogeneity (I^2^ = 73.17%, τ^2^ = 2.071; Q(28) = 104.36, *p* < 0.001), whereas no statistical heterogeneity was observed in the PNF cohort (I^2^ = 0.0%, τ^2^ = 0.000; Q(13) = 12.22, *p* = 0.509). The heterogeneity observed among CCH cohorts is likely multifactorial, reflecting differences in study design, cohort size, follow-up duration, denominator definition, injection and manipulation protocols, and methods of adverse-event ascertainment. In particular, large post-marketing surveillance datasets differ substantially from prospective clinical cohorts in both exposure estimation and event capture. The large post-marketing CCH analysis by Peimer et al. [20] contributed most rupture events but reported a comparatively low treatment-level incidence, whereas smaller CCH cohorts produced higher point estimates with wider confidence intervals. Additional subgroup analysis or meta-regression according to these factors was considered; however, rupture events were sparse and further stratification would have resulted in small groups dominated by zero-event studies. Such analyses would therefore have produced unstable estimates with limited interpretability. A qualitative exploration of potential sources of heterogeneity was considered more appropriate.

Previous comparative literature and systematic reviews have similarly suggested that tendon rupture may occur more frequently following CCH than PNF, although absolute event rates remain low [5,9,14,19]. In this review, crude reported incidences ranged from 0.09% to 1.7% for CCH and from 0.12% to 0.21% for PNF. The pooled estimates therefore support the broader observation that flexor tendon rupture is rare after both treatments but may be more frequently reported following CCH.

Differences in complication profiles may relate to the distinct mechanisms of each treatment. CCH acts through enzymatic degradation of collagen within Dupuytren cords, whereas PNF mechanically divides the cord using a needle [8,20,22,33,34,35,36]. Inadvertent exposure of adjacent collagen-containing structures during CCH injection has been proposed as a mechanism of tendon injury [15,32]. Injections placed too deeply or too close to the flexor sheath may increase collagenase exposure to adjacent tendon structures, potentially predisposing to rupture [1,23,32,37]. By contrast, tendon injury during PNF is generally thought to occur through direct mechanical trauma from the needle [7,36]. Accordingly, the risk profile of PNF may depend more heavily on operator experience, procedural technique, and accurate assessment of tendon depth and anatomy.

Several potential risk factors have been proposed, including severe proximal interphalangeal joint (PIPJ) contracture, recurrent disease, previous intervention, and small-finger involvement [18,19,34]. Severe or recurrent disease may distort normal tissue planes and reduce the separation between the Dupuytren cord and adjacent flexor tendons, increasing susceptibility to iatrogenic injury [23,32]. In this review, the small finger was the most frequently affected digit, followed by the ring finger. However, this may partly reflect the underlying distribution of Dupuytren’s disease, which commonly affects the ulnar digits, rather than a true digit-specific rupture risk [2,22,37].

Among reported cases, rupture most commonly involved the FDP tendon, either in isolation or in combination with FDS. The median time to rupture was 8 days, with most reported cases occurring within 2 weeks of treatment. Delayed ruptures were also identified following CCH, including cases at 3 months, 106 days, 112 days, and 4 months. These delayed presentations suggest that tendon rupture may not always represent an immediate procedural complication and may instead reflect progressive weakening, secondary procedures, or subsequent mechanical stress in a vulnerable digit.

Management was most commonly surgical when reported, including tendon excision, tendon grafting, reconstruction, and tenorrhaphy. This complication is clinically important because it commonly affects the small and ring fingers, which are also frequently involved in DD [37]. Surgical reconstruction in this setting may be technically challenging due to pre-existing fibrosis, recurrent disease, altered tissue planes, and stiffness, all of which may limit restoration of optimal hand function [23].

Several authors have proposed ultrasound-guided CCH injection and PNF as potential strategies to improve procedural safety. Ultrasound allows real-time visualisation of the Dupuytren cord, flexor tendons, and adjacent neurovascular structures, which may be particularly useful in recurrent disease, severe contracture, or anatomically distorted digits [54,55,56]. For CCH injection, careful attention to injection depth, cautious manipulation, and awareness of high-risk anatomical regions such as the small-finger PIPJ may theoretically reduce the likelihood of tendon injury [19,22]. However, evidence supporting these preventative strategies remains limited, and prospective studies are required to determine whether ultrasound guidance meaningfully reduces severe complications such as flexor tendon rupture.

From a clinical perspective, these findings may assist informed consent and shared decision-making by providing an estimate of the absolute frequency of a rare but potentially functionally significant complication. Flexor tendon rupture should be presented to patients as uncommon following both CCH and PNF, and the present data should not be interpreted as establishing that one treatment is definitively safer than the other. Rather, counselling may incorporate the differing mechanisms of injury: enzymatic weakening of adjacent flexor structures following CCH and direct mechanical injury during PNF. Treatment selection should continue to account for disease distribution and severity, particularly PIPJ and small-finger disease, previous intervention or recurrent disease, anticipated recurrence, recovery requirements, treatment availability, operator experience, and patient preference. The low absolute incidence observed in this review suggests that flexor tendon rupture alone should not determine treatment selection, but its potentially substantial functional consequences warrant inclusion in informed consent.

These observations also have potential relevance to personalised medicine in Dupuytren’s disease. Future risk-stratification approaches may integrate treatment modality with patient- and disease-specific characteristics, including the affected digit and joint, contracture severity, recurrent disease, previous intervention, and local tissue anatomy. Such models could ultimately support more individualised estimates of procedural risk and treatment selection; however, the present data are insufficient to establish or validate a patient-level prediction model.

### 4.2. Limitations

This study has several limitations. Flexor tendon rupture is a rare adverse event, and most included studies were not designed to evaluate it as a primary outcome. Consequently, rupture data were often derived from secondary adverse-event reporting, with limited patient-level detail regarding timing, tendon involvement, disease severity, prior treatment, affected digit or joint, and management.

An additional consideration is that the denominator units used across studies do not represent identical measures of risk. Patient-level denominators may include individuals undergoing treatment of multiple digits, whereas finger-, digit- or ray-level denominators more closely approximate the anatomical unit at risk of tendon rupture. Treatment-level denominators may additionally include repeated interventions in the same patient or digit. Consequently, the same number of rupture events may produce different incidence estimates depending on the unit reported. Because the primary studies generally did not provide sufficient information to convert these denominators reliably to a common unit, we preserved the original study-level denominator and prioritised finger/digit-level data where available. This methodological heterogeneity likely contributes to between-study variability and means that the pooled estimates should be interpreted as approximate incidences across reported treatment units rather than as uniform per-patient risks.

Furthermore, reporting bias remains an inherent limitation in the literature. Rare adverse-event outcomes are particularly susceptible to reporting and ascertainment bias. Prospective trials are generally not powered to identify uncommon complications such as tendon rupture, while retrospective cohorts depend on the completeness of clinical documentation and follow-up. Post-marketing surveillance introduces additional limitations because adverse events may be reported spontaneously, event capture may be incomplete, clinical details may be unavailable, and treatment exposure may be estimated rather than recorded at the individual-patient level. Conversely, unusual or severe complications may be preferentially reported or published, particularly in smaller clinical series. The direction and magnitude of these biases are therefore difficult to predict. The markedly different incidence estimates observed between large post-marketing datasets and smaller clinical cohorts should consequently be interpreted in the context of their differing ascertainment methods rather than as directly comparable estimates of biological risk.

Finally, postmarketing surveillance studies may be affected by reporting bias due to spontaneous and incomplete adverse-event reporting, while smaller clinical studies may produce unstable incidence estimates because single events have a large proportional effect.

## 5. Conclusions

Flexor tendon rupture is an uncommon but clinically important complication following minimally invasive treatment of Dupuytren’s disease. In this meta-analysis, pooled rupture incidence was higher following CCH than PNF, at 0.83% versus 0.32%, respectively, with a statistically significant subgroup difference. However, this should be interpreted cautiously given sparse events, heterogeneous denominators, and limited direct comparative evidence.

The observed difference may reflect the distinct mechanisms of each treatment, with CCH-related rupture potentially related to enzymatic weakening of adjacent flexor structures and PNF-related rupture more likely due to direct mechanical injury. Further prospective studies using standardised digit-level reporting are needed to clarify risk factors and assess whether strategies such as ultrasound guidance and procedural refinement can reduce this rare but significant complication.

Future prospective studies incorporating standardised patient, anatomical, disease, and procedure level variables may enable individualised risk stratification and support a more personalised approach to treatment selection in Dupuytren’s disease.

## Figures and Tables

**Table 1:** Characteristics of the studies included in this review.

	*n*	Mean (SD)
Included studies	41	
CCH only	20	
CCH vs. PNF	8	
PNF only	8	
CCH vs. placebo	2	
CCH vs. fasciectomy (limited or open)	2	
PNF vs. fasciectomy (limited or open)	1	
Procedure groups	55	
CCH injections	33	
PNF	17	
Fasciectomy (limited or open)	3	
Placebos	2	
Patients (treated cohort based on 49 procedure groups)	7708	
CCH (based on 32 procedure groups reporting patient-level data)	3649	
PNF	4059	
Gender, male (based on 44 procedure groups)	5563	
Mean age, in years (based on 46 procedure groups)		65.5 (2.4)
Follow up, in months (based on 55 procedure groups)		19.6 (26.0)

**Table 2:** Characteristics of reported flexor tendon rupture cases from included studies.

	*n*	Median
Total cases of flexor tendon rupture	68	
Cases of flexor tendon rupture as a complication of CCH treatment	62	
Cases of flexor tendon rupture as a complication of PNF treatment	6	
Types of tendon rupture		
Isolated FDP ^1^ rupture	16	
Isolated FDS ^2^ rupture	4	
Combined FDP + FDS rupture	7	
Not reported	41	
Joints affected		
MCPJ ^3^	23	
PIPJ ^4^	20	
PIPJ + MCPJ	3	
Not reported	22	
Fingers affected		
Small finger	32	
Ring finger	12	
Middle finger	5	
Index finger	3	
Not reported	16	
Timing to rupture, in days (based on 33 cases)		8 days
Management		
Surgical repair	11	
Non-surgical	1	
No management	1	
Not reported	55	

^1^ Flexor digitorum profundus; ^2^ flexor digitorum superficialis; ^3^ metacarpophalangeal joint; ^4^ proximal interphalangeal joint.

## Data Availability

All data is publicly available.

## Supplementary Materials

The following supporting information can be downloaded at https://www.mdpi.com/article/10.3390/jpm16090459/s1, Table S1: Summary of risk of bias assessment for included studies; Table S2: Data extracted from included studies. Table S3: PRISMA_2020_checklist.

## Abbreviations

The following abbreviations are used in this manuscript:

CCH	Collagenase *Clostridium histolyticum*
CI	Confidence interval
DD	Dupuytren’s disease
FDP	Flexor digitorum profundus
FDS	Flexor digitorum superficialis
JBI	Joanna Briggs Institute
MCP	Metacarpophalangeal
MCPJ	Metacarpophalangeal joint
MeSH	Medical Subject Headings
NOS	Newcastle-Ottawa Scale
PIP	Proximal interphalangeal
PIPJ	Proximal interphalangeal joint
PNA	Percutaneous needle aponeurotomy
PNF	Percutaneous needle fasciotomy
PRISMA	Preferred Reporting Items for Systematic Reviews and Meta-Analyses
RCT	Randomised controlled trial
RoB 2	Cochrane Risk of Bias 2 tool
SD	Standard deviation
