# Supplementary material for: Flexor Tendon Rupture Following Collagenase Clostridium histolyticum Versus Percutaneous Needle Fasciotomy in the Management of Dupuytren’s Disease: A Systematic Review and Meta-Analysis
Source: J Pers Med. 2026 Aug 31;16(9):459. doi: 10.3390/jpm16090459 (PMC13608378; doi:10.3390/jpm16090459)
Supplement: Supplementary file 1 [file jpm-16-00459-s001.zip › Supplementary Table S1.pdf]

Table S1. Summary of risk of bias assessment for included studies

Study	Study design	Assessment tool	Overall bias rating
Hurst et al (2009) [8]	RCT	RoB 2	Low risk
Gilpin et al (2010) [34]	RCT	RoB 2	Low risk
Mickelson et al (2014) [35]	RCT	RoB 2	Some concerns
Strömberg et al (2016) [12]	RCT	RoB 2	Low risk
Skov et al (2017) [11]	RCT	RoB 2	Some concerns
Hauksson et al (2024) [22]	RCT	RoB 2	Low risk
Badalamente M.A, Hurst L.C (2000) [36]	Randomised clinical trial	RoB 2	Some concerns
Pess et al (2012) [17]	Retrospective cohort	NOS	Moderate
McMahon et al (2013) [32]	Retrospective cohort	NOS	Moderate
Nash H. Naam (2013) [37]	Retrospective cohort	NOS	Moderate
Nydick et al (2013) [23]	Retrospective cohort	NOS	Moderate
Medjoub K., Jawad A. (2014) [18]	Retrospective cohort	NOS	High
Herrera et al (2015) [24]	Retrospective cohort	NOS	Moderate
Scherman et al (2016) [53]	Retrospective cohort	NOS	Moderate
Zhou et al (2016) [33]	Retrospective cohort	NOS	Moderate
Grandizio et al (2017) [38]	Retrospective cohort	NOS	Moderate
Hwee et al (2017) [39]	Retrospective cohort	NOS	Moderate
Molenkamp et al (2017) [25]	Retrospective cohort	NOS	Moderate
Cook et al (2019) [40]	Retrospective cohort	NOS	Moderate
Abdelrahman et al (2020) [54]	Retrospective cohort	NOS	Moderate
Patel M.I.A, Patel I.A (2020) [26]	Retrospective cohort	NOS	Moderate
Therkelsen et al (2020) [7]	Retrospective cohort	NOS	Low
Mehdiyev et al (2022) [27]	Retrospective cohort	NOS	Moderate
Rahr et al (2011) [9]	Prospective cohort	NOS	Moderate

Gaston et al (2015) [41]	Prospective cohort	NOS	Low
Arora et al (2016) [42]	Prospective cohort	NOS	Moderate
Verstreken et al (2016) [43]	Prospective cohort	NOS	Moderate
Hansen et al (2017) [44]	Prospective cohort	NOS	Moderate
Simón-Pérez et al (2018) [45]	Prospective cohort	NOS	Moderate
Werlinrud et al (2018) [46]	Prospective cohort	NOS	Moderate
Arora et al (2019) [47]	Prospective cohort	NOS	Low
Zachrisson et al (2020) [29]	Prospective cohort	NOS	Moderate
Zhou et al (2015) [48]	Propensity score-matched	NOS	Low
Coleman et al (2012) [49]	Open-label pilot study	NOS	High
Coleman et al (2014) [31]	Non-randomised clinical trial	NOS	Moderate
Hirata et al (2017) [50]	Non-randomised clinical trial	NOS	Moderate
Considine et al (2015) [51]	Case series	JBI	Moderate
Malafa et al (2015) [52]	Case series	JBI	Moderate
Peimer et al (2012) [30]	Postmarketing safety analysis	Narrative	Higher risk of reporting bias
Peimer et al (2022) [20]	Postmarketing safety analysis	Narrative	Moderate
