# Supplementary material for: Flexor Tendon Rupture Following Collagenase Clostridium histolyticum Versus Percutaneous Needle Fasciotomy in the Management of Dupuytren’s Disease: A Systematic Review and Meta-Analysis
Source: J Pers Med. 2026 Aug 31;16(9):459. doi: 10.3390/jpm16090459 (PMC13608378; doi:10.3390/jpm16090459)
Supplement: Supplementary file 1 [file jpm-16-00459-s001.zip › Supplementary Table S2.pdf]

Table S2. Data extracted from included studies

Author (year)	Study design	Procedure	Denominator for incidence calculation	Flexor tendon rupture (n)	Incidence of flexor tendon rupture (%)	Timing to rupture	Tendon ruptured	Joint/Digit affected	Management
*Badalamente M.A, Hurst L.C (2000) [36]	Randomised phase 2 clinical trial	CCH	35 patients	0	0	-	-	-	-
**Hurst et al (2009) [8]**	RCT	CCH vs placebo	204 patients	2	0.98	8 days	FDP[1]	PIPJ[2]/Small finger	Surgical repair - excision of FDP and tenolysis of FDS followed by physical therapy.
						8 days	FDP + FDS[3]	MCPJ[4]/Small finger	Excision of FDP + FDS and placement of a Hunter rod, followed by hand therapy and transplantation of tendon into FDP.
Gilpin et al (2010) [34]	RCT	CCH vs placebo	45 patients	0	0	-	-	-	-
*Coleman et al (2012) [49]	Open-label pilot study	CCH	12 patients	0	0	-	-	-	-
**Peimer et al (2012) [30]**	Post- marketing safety analysis	CCH	115 patients	2	1.7	2 weeks	FDP	PIPJ/Small finger	Tendon graft
						3 months	FDS	MCPJ/Ring finger	No corrective procedures were planned or

									undertaken at time of report
**McMahon et al (2013) [32]**	Retrospective cohort	CCH	102 patients	1	0.98	NR[5]	NR	PIPJ/Small finger	NR
*Nash H. Naam (2013) [37]	Retrospective cohort	CCH vs fasciectomy	21 fingers	0	0	-	-	-	-
**Coleman et al (2014) [31]**	Non-randomised clinical trial	CCH	60 patients	1	1.67	4 months	FDP + FDS	MCPJ/Ring finger	2-stage tendon reconstruction with graft for FDP rupture - during which FDS rupture was identified
Mickelson et al (2014) [35]	RCT	CCH	46 digits	0	0	-	-	-	-
Considine et al (2015) [51]	Case series	CCH	13 fingers	0	0	-	-	-	-
**Gaston et al (2015) [41]**	Prospective cohort	CCH	714 patients	1	0.14	3 days	FDP	PIPJ/Small finger	NR
Malafa et al (2015) [52]	Retrospective case series	CCH	36 patients	0	0	-	-	-	-
Zhou et al (2015) [48]	Propensity score matched cohort	CCH vs LF	66 patients	0	0	-	-	-	-
Arora et al (2016) [4]	Prospective cohort	CCH	120 patients	0	0	-	-	-	-
*Verstreken et al (2016) [43]	Prospective cohort	CCH	108 patients	0	0	-	-	-	-

Grandizio et al (2017) [38]	Retrospective cohort	CCH	34 digits	0	0	-	-	-	-
Hansen et al (2017) [44]	Prospective cohort	CCH	212 patients	0	0	-	-	-	-
Hirata et al (2017) [50]	Non-randomised phase 3 clinical trial	CCH	102 patients	0	0	-	-	-	-
Hwee et al (2017) [39]	Retrospective cohort	CCH	113 patients	0	0	-	-	-	-
*Simón-Pérez et al (2018) [45]	Prospective cohort	CCH	67 fingers	0	0	-	-	-	-
*Werlinrud et al (2018) [46]	Prospective cohort	CCH	104 patients	0	0	-	-	-	-
**Cook et al (2019) [40]**	Retrospective cohort	CCH	236 patients	1	0.42	3 days	FDP + FDS	PIPJ/Small finger	Single-stage FDP reconstruction with palmaris longus tendon graft
Arora et al (2019) [47]	Prospective cohort	CCH	788 patients	0	0	-	-	-	-
**Peimer et al (2022) [20]**	Post- marketing safety analysis	CCH	~57,416 treatments	54	0.09	Range from 1 to 26 days with delayed rupture at 112 and 106 days	FDP = 12<br>FDS = 2<br>FDP + FDS = 1<br>NR = 39	MCPJ = 20<br>PIPJ = 15<br>MCPJ + PIPJ = 3<br>NR = 16<br><br>Index finger = 3<br>Middle finger = 4	Majority was NR - only the case of delayed rupture of 106 days was reported to have undergone PIPJ arthrodesis and

								Ring finger = 10<br>Small finger = 26<br>NR = 11	repeat fasciectomy in that finger
Rahr et al (2011) [28]	Prospective cohort	PNF	213 rays	0	0	-	-	-	-
*Pess et al (2012) [17]	Retrospective cohort	PNF	1,013 fingers	0	0	-	-	-	-
Medjoub K., Jawad A. (2014) [18]	Retrospective cohort	PNF	63 fingers	0	0	-	-	-	-
**Herrera et al (2015) [24]**	Retrospective cohort	PNF	525 digits	1	0.19	NR	FDP	NR/Middle finger	FDS to FDP tendon transfer
Zhou et al (2016) [33]	Retrospective cohort	PNF vs LF	78 patients	0	0	-	-	-	-
**Molenkamp et al (2017) [25]**	Retrospective cohort	PNF	470 rays	1	0.21	NR	NR	NR	Surgical intervention - no further details reported
Patel M.I.A, Patel I.A (2020) [26]	Retrospective cohort	PNF	118 rays	0	0	-	-	-	-
**Therkelsen et al (2020) [7]**	Retrospective cohort	PNF	3,331 fingers	4	0.12	3 days	FDS	NR	Surgical repair - tenorrhaphy
						7 days	FDP + FDS	NR	Surgical repair - tenorrhaphy
						8 days	FDP + FDS	NR	Surgical repair - tenorrhaphy

						9 days	FDP + FDS	NR	Nonsurgical management
Zachrisson et al (2020) [29]	Prospective cohort	PNF	58 fingers	0	0	-	-	-	-
*Nydick et al (2013) [23]	Retrospective cohort	CCH vs PNF	59 patients (29 CCH, 30 PNF)	0	0	-	-	-	-
*Scherman et al (2016) [53]	Retrospective cohort	CCH vs PNF	86 rays	0	0	-	-	-	-
*Strömberg et al (2016) [12]	RCT	CCH vs PNF	140 fingers (69 CCH, 71 PNF)	0	0	-	-	-	-
Skov et al (2017) [11]	RCT	CCH vs PNF	50 patients (29 CCH; 21 PNF)	0	0	-	-	-	-
Abdelrahman et al (2020) [54]	Retrospective cohort	CCH vs PNF	180 fingers	0	0	-	-	-	-
Mehdiyev et al (2022) [27]	Retrospective cohort	CCH vs PNF	40 fingers (13 CCH, 27 PNF)	0	0	-	-	-	-
Hauksson et al (2024) [22]	RCT	CCH vs PNF	80 patients (40 CCH; 40 PNF)	0	0	-	-	-	-

[1]Flexor digitorum profundus; [2]Proximal interphalangeal; [3]Flexor digitorum superficialis; [4]Metacarpophalangeal joint; [5]Not reported

*Studies extracted from screening reference lists of systematic reviews and meta-analyses, literature reviews and clinical trial analyses.
